# An Internet-Based Virtual Coach to Promote Physical Activity Adherence in Overweight Adults: Randomized Controlled Trial

**DOI:** 10.2196/jmir.1629

**Published:** 2012-01-26

**Authors:** Alice Watson, Timothy Bickmore, Abby Cange, Ambar Kulshreshtha, Joseph Kvedar

**Affiliations:** ^1^Center for Connected HealthPartners HealthCareBoston, MAUnited States; ^2^Harvard Medical SchoolBoston, MAUnited States; ^3^College of Computer and Information ScienceNortheastern UniversityBoston, MAUnited States

**Keywords:** Activity monitoring, pedometers, obesity, body mass index, telemedicine, telehealth

## Abstract

**Background:**

Addressing the obesity epidemic requires the development of effective, scalable interventions. Pedometers and Web-based programs are beneficial in increasing activity levels but might be enhanced by the addition of nonhuman coaching.

**Objectives:**

We hypothesized that a virtual coach would increase activity levels, via step count, in overweight or obese individuals beyond the effect observed using a pedometer and website alone.

**Methods:**

We recruited 70 participants with a body mass index (BMI) between 25 and 35 kg/m^2^ from the Boston metropolitan area. Participants were assigned to one of two study arms and asked to wear a pedometer and access a website to view step counts. Intervention participants also met with a virtual coach, an automated, animated computer agent that ran on their home computers, set goals, and provided personalized feedback. Data were collected and analyzed in 2008. The primary outcome measure was change in activity level (percentage change in step count) over the 12-week study, split into four 3-week time periods. Major secondary outcomes were change in BMI and participants’ satisfaction.

**Results:**

The mean age of participants was 42 years; the majority of participants were female (59/70, 84%), white (53/70, 76%), and college educated (68/70, 97%). Of the initial 70 participants, 62 completed the study. Step counts were maintained in intervention participants but declined in controls. The percentage change in step count between those in the intervention and control arms, from the start to the end, did not reach the threshold for significance (2.9% vs –12.8% respectively, *P* = .07). However, repeated measures analysis showed a significant difference when comparing percentage changes in step counts between control and intervention participants over all time points (analysis of variance, *P* = .02). There were no significant changes in secondary outcome measures.

**Conclusions:**

The virtual coach was beneficial in maintaining activity level. The long-term benefits and additional applications of this technology warrant further study.

**Trial Registration:**

ClinicalTrials.gov NCT00792207; http://clinicaltrials.gov/ct2/show/NCT00792207 (Archived by WebCite at http://www.webcitation.org/63sm9mXUD)

## Introduction

With 65% of US adults being overweight, and a third meeting the criteria for obesity [1], health professionals have been spurred to develop innovative strategies to address this epidemic. A major driver of obesity is inadequate physical activity [2], with only a quarter of US adults engaging in the recommended amount of weekly physical activity [3].

Motivational coaching, personalized feedback, goal setting, and patient education have been used successfully to bring about long-term changes in diet and activity [4,5]. Delivering these components via traditional means, however, such as face-to-face interactions with a clinician, is costly and difficult to scale up.

New technologies have shown promise as effective, accessible, and inexpensive solutions. Pedometers, wearable devices that capture step count, can increase activity levels by up to 2000 steps per day [6]. In addition, the use of the Internet to communicate information to individuals may allow population health interventions to be delivered, and widely disseminated, at relatively low cost [7-10].To date, several Internet-based interventions have demonstrated reductions in weight through a combination of self-monitoring, education, and motivational messaging [11-13].

Such interventions may be enhanced by the addition of a coach, to promote accountability and adherence to an exercise program. In the helping professions there is a well-documented association between the quality of the professional–client relationship and outcomes [14]. The psychotherapy literature describes this working alliance as the trust and belief that the helper and patient have in each other as team members in achieving a desired outcome [15,16].

Coaching need not be carried out face-to-face; indeed, formal and informal e-coaching models have been demonstrated to be beneficial in promoting activity and weight loss [17,18]. Furthermore, a coach need not be human; recent research into the use of embodied computer agents has shown that participants can successfully form a working alliance relationship with a nonhuman agent or, simply put, a virtual coach [19].

In our study we sought to understand the effectiveness of virtual coaching compared with the use of a pedometer and website alone in improving activity levels in overweight or obese participants. We hypothesized that use of a virtual coach would increase their activity levels, in the form of step count, beyond the effect observed using a pedometer and website alone.

## Methods

### Eligibility Criteria

Participants were between 20 and 55 years old (inclusive); had a body mass index (BMI) between 25 and 35 kg/m^2^ (inclusive); were fluent in spoken and written English; had a primary care physician; had access to a personal computer with an available USB port, speakers, and Internet access; and either answered no to all 7 questions on the Physical Activity Readiness Questionnaire (PAR-Q) or obtained written permission from their primary care physician to take part in the study. The PAR-Q is a validated screening tool designed to identify adults with medical problems that might preclude them from safely initiating an exercise regimen [20].

### Setting

Recruitment took place in Boston, Massachusetts, USA through advertisements in local newspapers, on a local website (Craigslist), at health care facilities, and through broadcast emails within the hospital email network. All study visits took place at Massachusetts General Hospital, Boston.

Recruitment commenced in June 2008 and took 3 weeks. Data were collected and analyzed in 2008. The study was reviewed and approved in July 2007 by the institutional review board of the Massachusetts General Hospital. This trial was registered on clinicaltrials.gov (NCT00792207).

### Interventions

At an initial visit, all potential participants were weighed and measured using a waist-high, stand-on bariatric scale (Detecto, capacity 600 × 0.2 lb; Cardinal Scale Manufacturing Company, Webb City, MO, USA) and a mechanical wall-mounted stadiometer, both maintained by Massachusetts General Hospital Biomedical Engineering. Following confirmation of eligibility and consent, the participant was randomly assigned to the intervention or control arm of the study.

**Figure 1:**
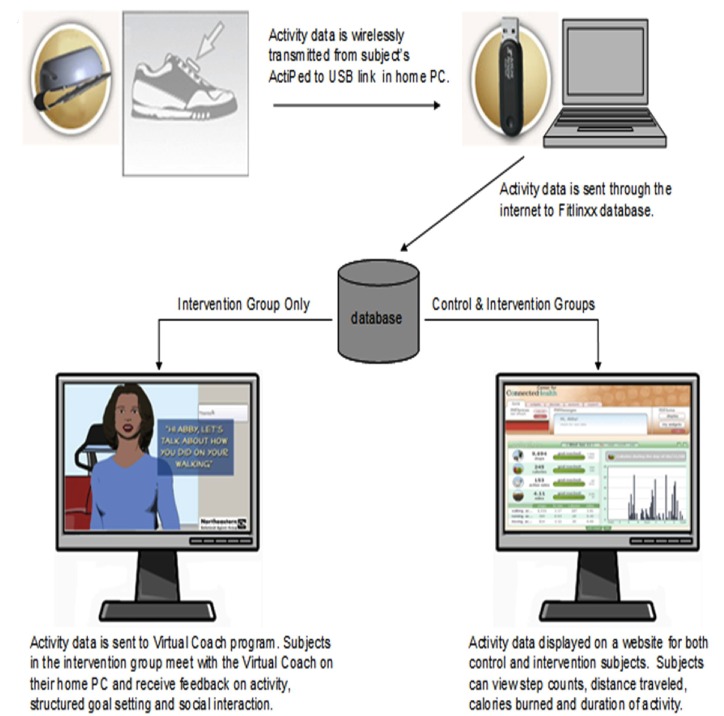
Illustration of technology used in the study and flow of data.

All participants were provided with the pedometer (ActiPed; FitLinxx, Norwalk, CT, USA) and instructed to wear it at all times over the 12-week study period, apart from when bathing or sleeping. The ActiPed device is a highly accurate activity monitor, worn on the shoe, which contains an accelerometer and tracks steps [21]. The device wirelessly transmits activity data to a USB receiver on a participant’s desktop computer, where it is then relayed over the Internet to a database on a secure computer server (Figure 1). All participants were given access to the pedometer manufacturer’s password-protected ActiHealth website to view graphs of their activity levels over time and set personal goals.

In addition, those in the intervention arm of the study were provided access to the virtual coach, a computer-animated exercise advisor that runs using software installed on users’ home computers. The virtual coach is entirely automated and follows an algorithm-driven script, using simulated face-to-face conversation, including verbal and nonverbal relationship-building behaviors modeled on best practices from studies of patient–provider health communication with the goal of establishing a working alliance [19]. The scripts used by the virtual coach were developed through an interdisciplinary collaboration involving physicians, computer scientists, and exercise trainers to ensure adherence to best practices. The script employs behavioral and social cognitive strategies demonstrated in the literature to promote exercise behavior change. These strategies include goal setting, shaping, self-monitoring, positive reinforcement, problem solving, education, and social support [22,23].

The virtual coach software was integrated with the database containing the participants’ activity data to allow tailored interactions according to each participant’s adherence to step count goals. The interactions all followed a structured pattern, starting with greeting and social interaction, proceeding to review of pedometer step count, feedback and goal setting, tips on activity or diet, and commitment to date of next interaction, and ending with encouragement and farewell. However, both the dialogue structure and the format and content of individual utterances were tailored based on each user’s progress in the system (eg, whether they had progressed past baseline), their current status (eg, whether they had met their short-term goals), and discourse context (eg, whether they had just asked the virtual coach a question or asked for help). As a result, those who had not met their activity target would have a different interaction at the same time point in the study from those who had met their goals. Users had to select from a series of answer options, as the system was not designed to handle free-text responses.

Software modules were significantly tested by the development team, followed by several end-to-end pilot tests of the intervention prior to deployment.

Intervention participants were instructed to meet with the coach three times a week throughout the study. These interactions lasted approximately 5 to 10 minutes per session. The 12-week program focused on building rapport and establishing baseline activity levels, followed by tips to increase activity, daily personalized goal setting, and advice about maintaining a healthy diet and activity level after the study concludes.

To ensure compliance and minimize loss of data throughout the 12-week study period, study staff contacted participants either if no step data were received or if those in the intervention arm did not talk to the virtual coach for 7 consecutive days. At the final visit, participants were weighed and measured and asked to complete several surveys.

 Participants were provided with gift cards for attendance at each study visit. The technology was provided at no charge, although all participants were required to have a computer with Internet access.

### Primary and Secondary Outcomes

The primary end point of this study was the percentage change in step count between those in the intervention and those in the control arms, from the start of the study compared with the end of the study. To evaluate the percentage change in step count, we divided the 12-week study into four 3-week time periods (P1, P2, P3, P4). Secondary end points were changes in weight, BMI, 7-day physical activity recall, physical activity stage of change, self-efficacy and exercise benefits and barriers, and satisfaction with the program [24-27]. Satisfaction was measured using a combination of novel questions regarding the activity monitor and standardized questions from the Working Alliance Inventory to assess the strength of social bond between intervention participants and the virtual coach [16].

### Sample Size

This study was designed to detect a difference of 12 in the percentage change in step count between intervention and control participants from P1 to P4. This number was determined following a priori discussions with clinical experts and was thought to represent a clinically significant change in steps assuming a baseline of between 5000 and 7000 steps per day. To have an 80% power to detect such a difference, assuming a standard deviation of 16, a type I error level of .05, and a dropout rate of 20%, a total sample size of 70 was required.

### Randomization

Random numbers were generated (using Microsoft Excel; Microsoft Corporation, Redmond, WA, USA) and assigned control or intervention status on a 1:1 basis with a block size of four. Sealed, ordered envelopes were prepared by one investigator, who was not involved in participant enrollment, containing information about group assignment and opened only after the participant consented. Due to the nature of the intervention, study staff and participants were not blinded to group assignment over the course of the study.

### Statistical Analysis

We compared continuous outcomes between groups using a Student *t* test (for normally distributed outcomes) and the Wilcoxon rank sum test (for nonnormally distributed outcomes and rank measures). Differences in proportions between groups were compared by using chi-square tests or Fisher exact test when appropriate. All calculations were performed with SAS version 9.1 (The SAS system for Windows; SAS Institute, Cary, NC, USA). We calculated average step counts for each 3-week period of time (period) by dividing the total number of steps recorded in the period by the number of days data were received. Days with a recorded step count of <100 were noted as missing data for the day, as this low level of activity was more likely to reflect the ActiPed being carried in a bag or moved in a house than actually being worn. Participants with no data for a period were noted as missing for this period. The analysis was conducted both examining only those participants with data for each period and including those who had missing data points in one, or more than one, period. A 2-sided *P* value of .05 was considered statistically significant. Average values are represented as mean (SE) unless otherwise stated.

## Results

### Patient Demographics

**Figure 2:**
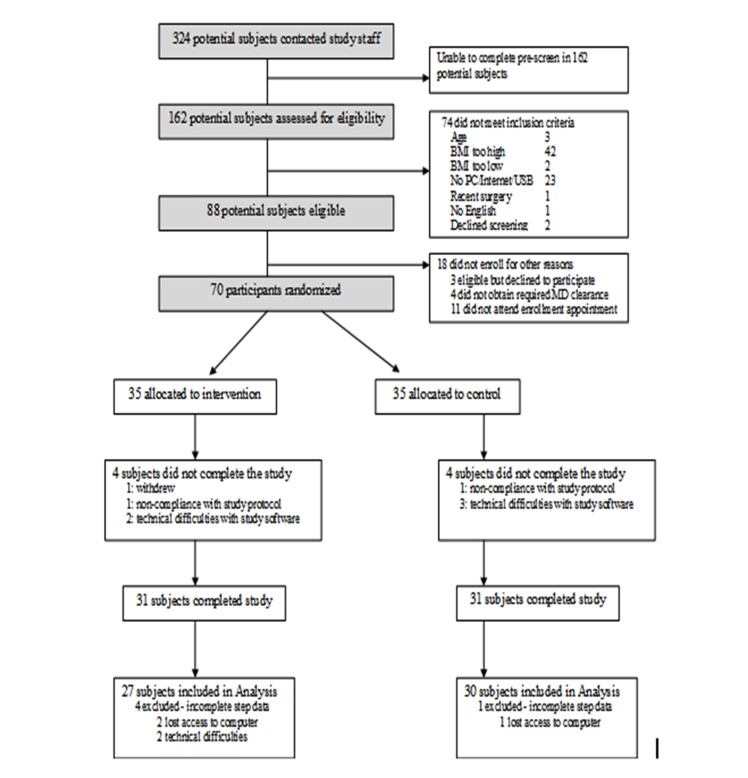
Flow diagram of participants' progress through the randomized controlled trial. BMI = body mass index; MD = medical doctor.

A total of 70 participants were enrolled, of whom 62 (89%) completed the study. The final participant completed the study in September 2008. Further details regarding enrollment are provided in Figure 2.

Participants were predominantly female(59/70, 84%), white (53/70, 76%), and college educated (68/70, 97%). Detailed baseline demographic information is reported in Table 1. There were no significant differences in age or baseline BMI between those who completed and those who did not complete the study.

**Table 1:** Baseline demographics of study participants

		Control (n = 35)	Intervention (n = 35)	*P* value
Female gender, n (%)		28/35 (80)	31/35 (89)	.51
Mean/median age (years)		40.6/41	44.1/9	.11
White race, n (%)		25/35 (71)	28/35 (80)	.57
Mean/median weight (kg)		84.1/82.4	81.3/81.7	.29
Mean/median BMI^a^ (kg/m^2^)		30.4/29.7	30.2/30.1	.79
College, n (%)		34/35 (97)	34/35 (97)	1.00
Income >US $50,000, n (%)		21/35 (62)	26/35 (74)	.31
Smoker, n (%)		2 (6)	2 (6)	1.0
**Surveys (median scores)**				
	7-day activity (kcal/day)	3071	2890	.13
	Self-efficacy	18.9	18.9	.94
	Benefits and barriers	136.6	137.1	.88
	Social support	35.4	34.6	.62
**Technology, n (%)**				
	Ever used a pedometer	18/35 (51)	14/35 (41)	.47
	Use Internet for information on exercise	29/35 (83)	21/35 (62)	.06
	Use Internet for information on diet	30/35 (86)	26/35 (77)	.37
	Ever used an online fitness program	8/35 (23)	10/35 (29)	.78
**Comorbidities, n (%)**				
	Hypercholesterolemia	6/35 (17)	5/35 (14)	1.0
	Hypertension	3/35 (9)	5/35 (14)	.70
	Asthma	4/35 (11)	4/35 (11)	1.0

^a^ Body mass index.

### Outcomes

**Figure 3:**
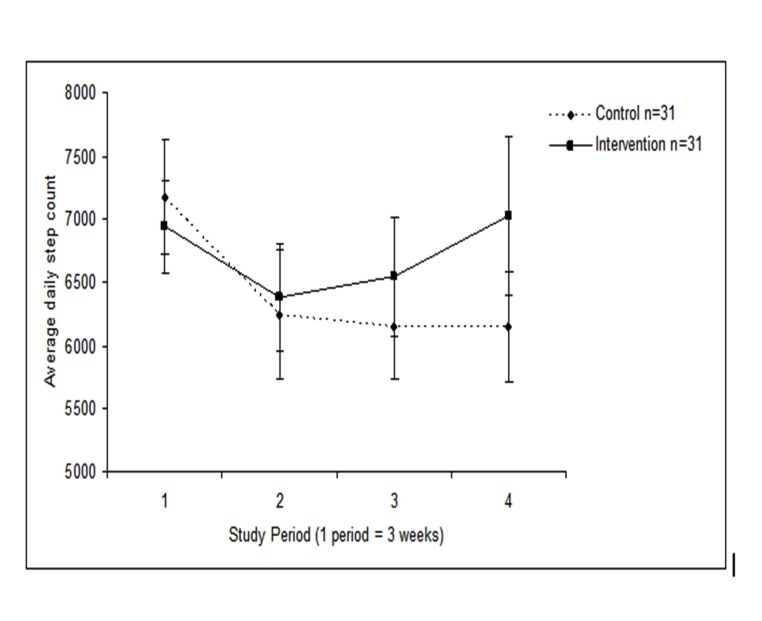
Average (SE) daily step count per period. Data shown for all participants who completed the study, including those with missing data.

Figure 3 shows the average step count per period according to group. The average step count in the control group fell significantly from 7174 in P1 to 6149 in P4 (*P* = .01). In contrast, the intervention participants’ mean step count did not change significantly from P1 (6943) to P4 (6943 vs 7024, respectively, *P* = .85).

**Figure 4:**
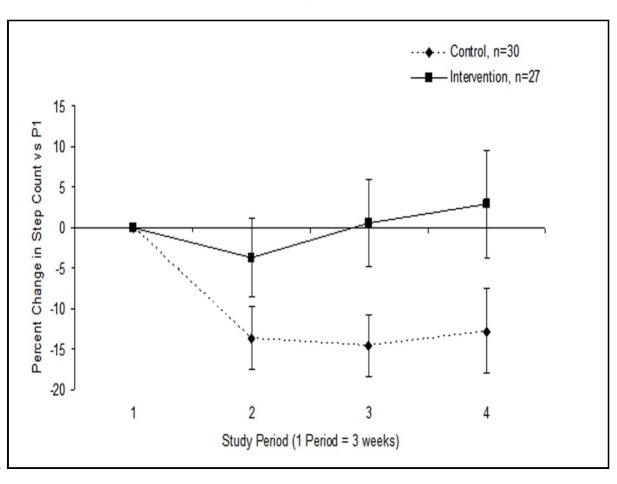
Average (SE) percentage change in step count in each period relative to the first time period (P1) for participants with complete data (ie, no missing periods).

Figure 4 charts the percentage change in mean activity levels for each period relative to period 1, only including participants with data for each period (30 control and 27 intervention). The difference seen between groups between P1 and P3 was statistically significant (*P* = .02), whereas the differences between P1 and P2, and between P1 and P4 did not meet criteria for significance (*P* = .12 and .07, respectively). A repeated measures analysis of variance, incorporating all data points from this figure, demonstrated that the percentage change in step count across all study periods was significantly different in the intervention versus control arms (*P* = .02).

We repeated this analysis including those participants who had data missing from one or more of the periods (eg, all completers). The changes from P1 to P2, P3, and P4 were not significant in this analysis (*P* = .14, .18, and .07 respectively). Due to the shifting sample size in this analysis it was not possible to perform a repeated measures analysis of variance on these data.

There were no statistically significant changes in secondary outcome measures. Mean reduction in BMI was 0.04 and 0.25 in control and intervention participants respectively (*P* = .44)

### Participants’ Adherence to Protocol

The mean number of days that step data were recorded over the course of the study was 73/84 (87%) for the control group and 71/84 (85%) for the intervention group (*P* = .64). Intervention participants had a mean number of sessions per participant over the course of the study of 28.9 (range 3–63, recommended 36). The mean number of visits per week fell from 2.8 in week 1 to 1.9 in week 12, although this change was not statistically significant (*P* = .08). There was no significant correlation between the number of sessions intervention participants had with the coach and their performance, in terms of either absolute step increase, absolute step count, or slope of step count change during the intervention.

### Participant Benefits and Satisfaction

Both intervention and control participants reported having benefited from taking part in the study (28/30, 93% and 28/31, 90% respectively, *P* = .67). Self-reported changes by intervention and control participants included exercising more frequently (25/29, 86% vs 21/29, 72%, *P* = .19) and improved diet and eating habits (13/29, 45% vs 6/29, 21%, *P* = .05), respectively.

Intervention participants were asked specific questions regarding their interactions with the virtual coach: 18/31 (58%) agreed that the coach motivated them to become more active and 27/31 (87%) reported feeling guilty if they skipped an appointment with the coach.

## Discussion

In this trial, providing overweight participants with access to a virtual coach in addition to a pedometer and website appeared to sustain step count over the course of the 12-week study, while step counts in control participants decreased from the start to the end. The percentage change in step count between those in the intervention and those in the control arms, from the start of the study compared with the end of the study, did not reach the threshold for significance (*P* = .07). However, repeated measures analysis found a significant difference when comparing percentage changes in step counts between control and intervention participants over all time points (*P* = .02).

These findings suggest that virtual coaching may be a useful adjunct to existing automated applications designed to promote activity, such as pedometers and online Web programs, and affects participant behavior over and above physiologic monitoring and text- and graph-based feedback. The virtual coach provides an interactive relationship, which is absent in the use of a pedometer or website alone. Previous research has shown that participants can form a social bond with a computer agent, even though they are aware that the agent does not represent an actual human [14]. We observed a somewhat dichotomous response to the virtual coach with some participants reporting negative emotions, such as guilt, anger, or frustration. Some of these emotions may actually promote activity, with the coach functioning as an external conscience or source of accountability, whereas others may erode the effectiveness of the relationship. Although we were not able to demonstrate significant difference in activity based on participants’ reactions to the coach, the observed trend suggests that dissatisfaction negatively affects the ability of the coach to promote increased activity. Further investigation into which segments of the patient population are most responsive to this form of automated coaching would allow us to maximize the impact of this intervention.

Figure 4 depicts a decline in activity in controls at the end of the study relative to their activity over the first 3 weeks. It appears that, rather than comparing which interventions increase activity by the largest amounts over extended time periods, interventions should be measured in terms of how well they promote sustained activity over time. Study participants, like the general population, are likely to be most motivated when commencing an activity regimen; maintaining this commitment over time is the challenge. Our study demonstrates that the decline in activity observed in control participants may be overcome through virtual coaching over a 12-week time period. In fact, the mean daily step counts in the last 3 weeks of the study were around 800 steps higher in intervention participants than in control participants, although this difference was not statistically significant. Given that pedometers have been demonstrated to increase step count by around 2000 steps per day [6], this suggests that virtual coaching might offer an additional increase in steps compared with pedometers alone. Longer study would be required to assess the effectiveness of virtual coaching over extended time periods.

Developing effective, automated self-management programs, that offer a relationship and personalized feedback, may prove essential to developing scalable solutions to deal with large populations faced with chronic disease. Technology-based solutions are becoming increasingly feasible as the proportion of Americans with access to cell phones and the Internet rises: current adoption estimates are 85% and 79%, respectively [28]. Internet-based health behavior-change programs may have more potential in the area of weight management than in many other health-related areas [29], as obese people are more likely to participate in follow-up because of a personal and nonstigmatizing approach via the Internet. Internet-based physical activity and weight-management programs have been shown to promote behavior change [30], particularly in occupational health settings [31], but the results haven’t always been consistent. A randomized trial of an Internet-based weight-management intervention among military personnel had shown benefits by preventing weight gain [32]. Studies should, however, assess the quality and appropriateness of the tailored advice. For example, a study by Slootmaker et al [33] evaluated the feasibility and effectiveness of a 3-month intervention in which office workers were provided with a personal activity monitor coupled to simple and concise Web-based tailored physical activity advice. They observed no significant intervention effect in the physical activity outcomes at the 8-month follow-up. Whether Internet-based interventions are more effective in helping people sustain their weight loss than in promoting new weight loss is an area of active inquiry [34].

Equitable access to technology is still a further concern, as groups most affected by obesity, such as minorities and low-socioeconomic status groups, are least likely to have access to technology [35]. Even low-cost, text messaging-based interventions delivered on cell phones have shown positive short-term behavioral outcomes [36]. Therefore, providing access to computers at local libraries or creating coaching applications that run on cell phones may be helpful strategies to bridge this divide [37-39].

This application may also be of value in tackling weight and activity issues among the pediatric or adolescent population. These groups are avid users of new technology and may be receptive to automated Web-based programs. To reduce the prevalence of overweight and obesity in these populations, it may also be necessary to decrease time spent in sedentary behaviors, such as leisure-time Internet and computer use [40]. Previous projects in these populations have primarily focused on school-based interventions, although the need to develop and evaluate Internet-based approaches has been flagged as a priority area for research [41].

There were several limitations to this study. Our study participants were primarily white, college-educated women. As a result, it may be difficult to generalize our findings to the wider population of overweight or obese patients who may be less comfortable taking a more active role in managing their health or in using technology. We do not have access to baseline step counts for study participants. We did, however, survey participants about baseline activity levels and found no significant difference. The step counts observed in the first few weeks after enrollment likely reflect an increase from baseline step counts for both intervention and control participants. It is also difficult to compare the results of this intervention with other pedometer-based programs because, unlike many commercially available pedometers, the ActiPed does not give participants immediate feedback on their current step count.

The length of the study was 12 weeks: ideally benefits of an activity or weight-loss program would be assessed over a longer time period. If this study were to be repeated over a longer time period, the coaching algorithm would need to be expanded to allow for a more variable series of interactions to maintain participants’ interest. Some of our participants reported finding the coach repetitive even over a 12-week time period. Conducting the study over a longer period of time, or with more participants, would allow for more robust assessment of highly relevant secondary outcome measures, such as decrease in BMI.

Finally, study staff contacted both intervention and control participants if they were noncompliant with the use of the pedometer or virtual coach, or if they were experiencing technical difficulties. There was more contact with the intervention participants (51 calls) than with control participants (31 calls). This likely reflects the fact that the intervention group had two different technologies to operate. This difference in contact may have had some bearing on the observed effects over the course of the study, but we think this is likely to be minor, as contact was not related to level of step count.

Virtual coaching has many applications beyond promoting activity. Coaching is increasingly recognized as an important component in the management of chronic conditions, such as diabetes and heart disease, and in the promotion of healthy behaviors, such as adherence to medication [42]. Given the growing burden of chronic disease and the shortage of providers [43], such applications may prove useful adjuncts to conventional office-based care. By linking data from home monitoring devices such as scales, blood pressure cuffs, or glucometers to automated coaching programs, health practitioners could encourage patients to develop better self-management skills. Clinician input could be provided if predefined triggers were alerted. Physicians have been slow to adopt information technology tools [44] due to concerns around reimbursement, data overload, and clinical outcomes, so thoughtful integration within existing workflow, including electronic medical record integration, would be necessary to ensure participation.

Broader changes in the health care payment system, such as the shift from visit-based to outcome-based reimbursement, may promote adoption of this type of care-delivery platform by clinicians. Pay-for-performance initiatives promote provider innovation around care delivery. An online coaching platform could be used, either as an adjunct to traditional care or as a stand-alone self-management program for patients.

### Conclusion

In this study we demonstrated a sustained level of activity in overweight participants provided with a virtual coach in addition to a pedometer and Web-based feedback, compared with a decline seen in those provided with a pedometer and Web-based program alone. Further work should examine the long-term benefits of virtual coaching and the extension of this application to a wider patient population.

## Multimedia Appendix 1

CONSORT-EHEALTH V1.6 checklist [45].

## Abbreviations

BMI: body mass index
PAR-Q: Physical Activity Readiness Questionnaire
