# Supplementary material for: An Internet-Based Virtual Coach to Promote Physical Activity Adherence in Overweight Adults: Randomized Controlled Trial
Source: J Med Internet Res. 2012 Jan 26;14(1):e1. doi: 10.2196/jmir.1629 (PMC3374543; doi:10.2196/jmir.1629)
Supplement: Supplementary file 1 [file jmir_v14i1e1_app1.pdf]

CONSORT-EHEALTH Checklist V1.6 Report

Manuscript Number 1629

Date completed 8/25/2011 14:50:29

by Alice Watson

A Randomized Controlled Trial of an Internet-based Virtual Coach to Promote Physical Activity Adherence in Overweight Adults

TITLE

1a-i) Identify the mode of delivery in the title

Yes. “ A Randomized Controlled Trial of an Internet-based Virtual Coach to Promote Physical Activity Adherence in Overweight Adults ”

1a-ii) Non-web-based components or important co-interventions in title

Non-web-based components like the pedometer were given to all participants and not part of the specific intervention being tested in this study. Therefore such components were not relevant for the title.

1a-iii) Primary condition or target group in the title

Yes. “ A Randomized Controlled Trial of an Internet-based Virtual Coach to Promote Physical Activity Adherence in Overweight Adults ”

ABSTRACT

1b-i) Key features/functionalities/components of the intervention and comparator in the METHODS section of the ABSTRACT

Yes. Key features are reported in the methods section of abstract as follows:
“Participants were assigned to one of two study arms and asked to wear a pedometer and access a website to view step counts. Intervention subjects also met with a Virtual Coach, an automated, animated computer agent that ran on their home computers, set goals and provided personalized feedback.”

1b-ii) Level of human involvement in the METHODS section of the ABSTRACT

Yes "Intervention subjects also met with a Virtual Coach, an automated, animated computer agent that ran on their home computers, set goals and provided personalized feedback.”

1b-iii) Open vs. closed, web-based (self-assessment) vs. face-to-face assessments in the METHODS section of the ABSTRACT

Participants “were recruited from the Boston metropolitan area”. Further details are provided in the manuscript text -“Recruitment took place in Boston, Massachusetts through advertisements in local papers, on a local website (Craigslist), at healthcare facilities and through broadcast emails within the hospital email network. All study visits took place at Massachusetts General Hospital.”
This study had few ‘face-to-face’ components for initial assessment, ensuring compliance and secondary outcomes in the final visit. After the initial visit, “study staff contacted participants if either no step data was received, or if those in the intervention arm failed to talk to the Virtual Coach for 7 consecutive days. At the final visit, participants were weighed and measured and asked to complete a number of surveys”.

1b-iv) RESULTS section in abstract must contain use data

Yes. “The mean age of participants was 42 years; the majority were female (84%), white (76%) and college educated (97%). Sixty-two of the initial 70 participants completed the study. Step counts were maintained in intervention subjects but declined in control subjects. The percent change in step count between those in intervention and control arms, from the start to end did not reach the threshold for significance (+2.9% vs. -12.8% respectively, p=0.07). However, repeated measures analysis showed a significant difference when comparing percent changes in step counts between control and intervention participants over all time points (ANOVA p = 0.02). There were no significant changes in secondary outcome measures. ‘’

1b-v) CONCLUSIONS/DISCUSSION in abstract for negative trials

Yes. “The Virtual coach was beneficial in maintaining activity level."

INTRODUCTION

2a-i) Problem and the type of system/solution

Yes. Description of the problem: “only a quarter of US adults engaging in the recommended amount of weekly physical activity”
Yes. Description of the solution: “internet-based interventions have demonstrated reductions in weight through a combination of self-monitoring, education and motivational messaging” .The interventions described in the paper are intended for incorporation in broader healthcare program.
Goal of our specific intervention is to test the potential of Virtual Coach as an effective, accessible and relatively inexpensive and scalable solution for improving physical activity among overweight and obese people

2a-ii) Scientific background, rationale: What is known about the (type of) system

Yes. Scientific background: “Several internet-based interventions have demonstrated reductions in weight through a combination of self-monitoring, education and motivational messaging”
Rationale for this study: “recent research into the use of embodied computer agents has shown that participants can successfully form a working alliance relationship with a non-human agent, or simply put a virtual coach.” Our objective is “to understand the effectiveness of virtual coaching compared to the use of a pedometer and website alone in improving activity levels in overweight or obese participants.”

METHODS

3a) CONSORT

Yes. “We hypothesized that use of a Virtual Coach would increase their activity levels, in the form of step count, beyond the effect observed using a pedometer and website alone.”

3b-i) Bug fixes, Downtimes, Content Changes

The technology was thoroughly piloted prior to commencing the RCT therefore no major bugs or downtime was encountered.

4a-i) Computer / Internet literacy

Yes. Participants “had access to a personal computer with an available USB port, speakers and Internet access” In addition we assessed computer literacy by asking about experience with relevant online activities e.g. searching for diet / exercise info on the Internet or participating in an online diet program.

4a-ii) Open vs. closed, web-based vs. face-to-face assessments:

Participants “were recruited from the Boston metropolitan area”. Further details are provided in the manuscript text -“Recruitment took place in Boston, Massachusetts through advertisements in local papers, on a local website (Craigslist), at healthcare facilities and through broadcast emails within the hospital email network. All study visits took place at Massachusetts General Hospital.”
This study had few ‘face-to-face’ components for initial assessment, ensuring compliance and secondary outcomes in the final visit. After the initial visit, “study staff contacted participants if either no step data was received, or if those in the intervention arm failed to talk to the Virtual Coach for 7 consecutive days. At the final visit, participants were weighed and measured and asked to complete a number of surveys”.
Both study staff and participants were unblinded over the course of the study.

4a-iii) Information giving during recruitment

All participants were provided with IRB-approved consent form outlining both arms of the study (included as appendix). Sealed, ordered envelopes were prepared containing information about group assignment and only opened after the participant had provided informed consent.

4b-i) Report if outcomes were (self-)assessed through online questionnaires

Primary outcome of the study (average step count) was assessed by wireless transmission of activity data to a USB receiver on the participant’s computer from where it is then relayed over the internet to a database on a secure computer server. At the final visit, participants were weighed and measured and asked to complete a number of surveys (secondary outcomes)

4b-ii) Report how institutional affiliations are displayed

5-i) Mention names, credential, affiliations of the developers, sponsors, and owners

The scripts used by the Virtual Coach were developed through an interdisciplinary collaboration involving physicians, computer scientists, and exercise trainers to ensure adherence to best practices. This was an academic collaboration without industry sponsors. A standard pedometer + website was provided to all participants.

5-ii) Describe the history/development process

Yes.“Significant testing of software modules was performed by the development team, followed by several end-to end pilot tests of the intervention prior to deployment.”

5-iii) Revisions and updating

5-iv) Quality assurance methods

Yes. “Significant testing of software modules was performed by the development team, followed by several end-to end pilot tests of the intervention prior to deployment.” Also,
“study staff contacted participants if either no step data was received, or if those in the intervention arm failed to talk to the Virtual Coach for 7 consecutive days.” This was done to ensure compliance and also to ascertain if the software was running satisfactorily.

5-v) Ensure replicability by publishing the source code, and/or providing screenshots/screen-capture video, and/or providing flowcharts of the algorithms used

Limited screen shots are provided in the paper

5-vi) Digital preservation

5-vii) Access

Yes. “Subjects were provided with gift cards for attendance at each study visit. The technology was provided at no charge, although all participants were required to have a computer with internet access.”

5-viii) Mode of delivery, features/functionalities/components of the intervention and comparator, and the theoretical framework

Yes. “The Virtual Coach is entirely automated and follows an algorithm-driven script; using simulated face-to-face conversation, including verbal and nonverbal relationship building behaviors modeled on best practices from studies of patient–provider health communication with the goal of establishing a working alliance. The scripts used by the Virtual Coach were developed through an interdisciplinary collaboration involving physicians, computer scientists, and exercise trainers to ensure adherence to best practices. The script employs a number of behavioral and social cognitive strategies demonstrated in the literature to promote exercise behavior change. These strategies include goal setting, shaping, self monitoring, positive reinforcement, problem solving, education and social support.”
“The Virtual Coach software was integrated with the database containing subject activity data to allow tailored interactions according to each subject’s adherence to step count goals. The interactions all followed a structured pattern, starting with greeting and social interaction, review of pedometer step count, feedback and goal setting, tips on activity or diet, commitment around date of next interaction, to encouragement and farewell. However, both dialogue structure and the format and content of individual utterances were tailored based on each user’s progress in the system (e.g., whether they had progressed past baseline or not), their current status (e.g., whether they had met their short-term goals or not), and discourse context (e.g., whether they had just asked the Virtual Coach a question or asked for help). As a result, those who had not met their activity target would have a different interaction at the same time point in the study from those who had met their goals. Users had to select from a series of answer options as the system was not designed to handle free text responses.”

5-ix) Describe use parameters

Yes. “Intervention participants were instructed to meet with the coach three times a week throughout the study. These interactions lasted approximately five to ten minutes per session. The 12-week program focused on rapport building and establishing baseline activity levels, followed by tips to increase activity, daily personalized goal setting, advice about maintaining a healthy diet and activity level after the study concludes.”

5-x) Clarify the level of human involvement

Yes. “throughout the 12-week study period, study staff contacted participants if either no step data was received, or if those in the intervention arm failed to talk to the Virtual Coach for 7 consecutive days.”

5-xi) Report any prompts/reminders used

Yes. “throughout the 12-week study period, study staff contacted participants if either no step data was received, or if those in the intervention arm failed to talk to the Virtual Coach for 7 consecutive days.” This was done to ensure compliance and if the software was working properly.

5-xii) Describe any co-interventions (incl. training/support)

There were no cointerventions in this study

6a-i) Online questionnaires: describe if they were validated for online use and apply CHERRIES items to describe how the questionnaires were designed/deployed

Yes. “Satisfaction was measured using a combination of novel questions regarding the activity monitor and standardized questions from the Working Alliance Inventory to assess the strength of social bond between intervention participants and the Virtual Coach.”

6a-ii) Describe whether and how “use” (including intensity of use/dosage) was defined/measured/monitored

Yes. “Intervention participants were instructed to meet with the coach three times a week throughout the study. These interactions lasted approximately five to ten minutes per session. The 12-week program focused on rapport building and establishing baseline activity levels, followed by tips to increase activity, daily personalized goal setting, advice about maintaining a healthy diet and activity level after the study concludes.”

6a-iii) Describe whether, how, and when qualitative feedback from participants was obtained

Yes. “Satisfaction was measured using a combination of novel questions regarding the activity monitor and standardized questions from the Working Alliance Inventory to assess the strength of social bond between intervention participants and the Virtual Coach.”

7a-i) Describe whether and how expected attrition was taken into account when calculating the sample size

Yes. “In order to have 80% power to detect such a difference, assuming standard deviation of 16, type 1 error level of 0.05 and a dropout rate of 20%, a total sample size of 70 was required.”

7b) CONSORT

No interim analyses were planned

8a) CONSORT

Yes. “Random numbers were generated (using Microsoft Excel) and assigned control or intervention status on a 1: 1 basis. Sealed, ordered envelopes were prepared containing information about group assignment and only opened after the participant consented.”

8b) CONSORT

Yes. 1:1 randomization scheme. Block size of 4.

9) CONSORT

Yes. “Random numbers were generated (using Microsoft Excel) and assigned control or intervention status on a 1: 1 basis. Sealed, ordered envelopes were prepared containing information about group assignment and only opened after the participant consented.”

10) CONSORT

"Random numbers were generated (using Microsoft Excel) and assigned control or intervention status on a 1: 1 basis with a block size of four. Sealed, ordered envelopes were prepared by one investigator, who was not involved in subject enrollment, containing information about group assignment and only opened after the participant consented."

11a-i) Specify who was blinded, and who wasn’t

Yes. “Due to the nature of the intervention, study staff and participants were not blinded to group assignment over the course of the study.”

11a-ii) Discuss e.g., whether participants knew which intervention was the “intervention of interest” and which one was the “comparator”

Yes. “Following confirmation of eligibility and consent, the participant was randomly assigned to the intervention or control arm of the study.” Participants knew if they were not randomized to the intervention-of-interest.

11b) CONSORT

Yes. “All participants were provided with the pedometer (ActiPed) and instructed to wear it at all times over the 12 week study period, apart from when bathing or sleeping.). … All participants were given access to the password-protected ActiHealth website to view graphs of their activity levels over time and set personal goals.”

12a) CONSORT

Yes. “Differences in proportions between groups were compared by using Chi-squared tests or Fisher's exact test when appropriate. All calculations were performed with SAS version 9.1 (The SAS system for windows. Cary NC: SAS institute inc. 1996). Average step counts for each 3 week period of time (period) were calculated by dividing the total number of steps recorded in the period by the number of days data was received…. A 2 sided p-value of 0.05 was considered statistically significant. Average values are represented as mean (standard error) unless otherwise stated."

12a-i) Imputation techniques to deal with attrition / missing values

Although no imputation techniques were done to deal with attrition/missing values, other measures were taken. “Days with a recorded step count of <100 were noted as missing data for the day, as this low level of activity was more likely to reflect the ActiPed being carried in a bag or moved in a house than actually being worn. Participants with no data for a period were noted as missing for this period. The analysis was conducted both examining only those participants with data for each period and including those who had missing data points in one, or more than one, period.”

12b) CONSORT

No adjusted or subgroup analyses was done.

RESULTS

13a) CONSORT
Yes. 'A total of 70 participants were enrolled of whom 62 (89%) completed the study. The final participant completed the study in September 2008. Further details regarding enrollment are provided in Figure 2."
13b) CONSORT
Yes. "Further details regarding enrollment are provided in Figure 2."
13b-i) Attrition diagram
No attrition figure included but drop-out included in figure 2.
14a) CONSORT
Yes. "Recruitment commenced in June 2008 and took a total of three weeks. Data were collected and analyzed in 2008."
14a-i) Indicate if critical "secular events" fell into the study period
None
14b) CONSORT
The study ended after complete follow-up mentioned apriori.
15) CONSORT
Yes. "Participants were predominantly female (84%), white (76%) and college educated (97%). Detailed baseline demographic information is reported in Table 1."
15-i) Report demographics associated with digital divide issues
Yes. "Participants were predominantly female (84%), white (76%) and college educated (97%).
16-i) Report multiple "denominators" and provide definitions
Yes. " Average step counts for each 3 week period of time (period) were calculated by dividing the total number of steps recorded in the period by the number of days data was received. Days with a recorded step count of <100 were noted as missing data for the day, as this low level of activity was more likely to reflect the ActiPed being carried in a bag or moved in a house than actually being worn. Participants with no data for a period were noted as missing for this period. The analysis was conducted both examining only those participants with data for each period and including those who had missing data points in one, or more than one, period. A 2 sided p-value of 0.05 was considered statistically significant. Average values are represented as mean (standard error) unless otherwise stated."

16-ii) Primary analysis should be intent-to-treat
Average step counts for each 3 week period of time (period) were calculated by dividing the total number of steps recorded in the period by the number of days data was received. Days with a recorded step count of <100 were noted as missing data for the day, as this low level of activity was more likely to reflect the ActiPed being carried in a bag or moved in a house than actually being worn. Participants with no data for a period were noted as missing for this period. The analysis was conducted both examining only those participants with data for each period and including those who had missing data points in one, or more than one, period. A 2 sided p-value of 0.05 was considered statistically significant. Average values are represented as mean (standard error) unless otherwise stated.

Given that step count was our primary outcome measure if this data was missing then participants could not be included in the analysis, however, we did included even those participatnets with some missing data for the time periods in which we did have readings.

17a) CONSORT
Yes. "The average step count in the control group fell significantly from 7174 in P1 to 6149 in P4 (P = 0.011). In contrast, the intervention participants' mean step count did not change significantly from P1 (6943) to P4 (6943 vs. 7024 respectively, P = 0.85)."
17a-i) Presentation of process outcomes such as metrics of use and intensity of use
Yes. "The mean number of days that step data were recorded over the course of the study was 73 (87%) for the control group and 71 (85%) for the intervention group (p=0.64). Intervention participants had a mean number of sessions per subject over the course of the study of 28.9 (range 3-63, recommended 36). The mean number of visits per week fell from 2.8 in week 1 to 1.9 in week 12 although this change was not statistically significant (P = 0.08). There was no significant correlation between the number of sessions intervention participants had with the coach and their performance, either in terms of absolute step increase, absolute step count, or slope of step count change during the intervention. "
17b) CONSORT
Yes. "The percent change in step count between those in intervention and control arms, from the start to end did not reach the threshold for significance (+2.9% vs. -12.8% respectively, p=0.07). However, repeated measures analysis showed a significant difference when comparing percent changes in step counts between control and intervention participants over all time points (ANOVA p = 0.02). There were no significant changes in secondary outcome measures."

18) CONSORT
No adjusted or subgroup analysis perfromed.
18-i) Subgroup analysis of comparing only users
Not done in this study.
19) CONSORT
No harm occurred to any participatn as a result of taking part in this study. This was a min imal risk study so we have not stressed this in the paper as harm was neither anticipiated nor encountered.
19-i) Include privacy breaches, technical problems
None occurred so not mentioned.
19-ii) Include qualitative feedback from participants or observations from staff/researchers
Yes. "Both intervention and control participants reported having benefited from taking part in the study (93.3% and 90.3% respectively, P = 0.67). Self-reported changes by intervention and control participants included exercising more frequently (86.2% vs. 72.4%, P = 0.19) and improved diet / eating habits (44.8% vs. 20.7%, P = 0.05) respectively.
Intervention participants were asked specific questions regarding their interactions with the Virtual Coach. 58.1% agreed that the coach motivated them to become more active and 87.1% reported feeling guilty if they skipped an appointment with the coach."

DISCUSSION

20-i) Typical limitations in ehealth trials
Yes. "Our study subjects were primarily white, college-educated women. As a result, it may be difficult to generalize our findings to the wider population of overweight or obese patients who may be less comfortable taking a more active role in managing their health or in utilizing technology. We do not have access to baseline step counts for study participants. We did, however, survey participants about baseline activity levels and found there to be no significant difference. It is likely that the step counts observed in the first few weeks after enrollment reflect an increase from baseline step counts for both intervention and control participants. It is also difficult to compare the results of this intervention with other pedometer-based programs because unlike many commercially available pedometers, the ActiPed does not give immediate feedback on current step count to participants.
The length of the study was 12 weeks: ideally benefits of an activity or weight loss program would be assessed over a longer time period. If this study was to be repeated over a longer time period the coaching algorithm would need to be expanded to allow for a more variable series of interactions to maintain subject interest. Some of our participants reported finding the coach repetitive even over a 12 week time period. Conducting the study over a longer period of time, or with more participants, would allow for more robust assessment of highly relevant secondary outcome measures, such as decrease in BMI.
Finally, study staff made contact with both intervention and control subjects if they were noncompliant with the use of the pedometer or Virtual Coach, or if they were experiencing technical difficulties. There was more contact with the intervention participants (51 calls) than with control participants (31 calls). This likely reflects the fact that the intervention group had 2 different technologies to operate. This difference in contact may have had some bearing on the observed effects over the course of the study but we think this is likely to be minor as contact was not related to level of step count."
21-i) Generalizability to other populations
Yes. "Our study subjects were primarily white, college-educated women. As a result, it may be difficult to generalize our findings to the wider population of overweight or obese patients who may be less comfortable taking a more active role in managing their health or in utilizing technology."
21-ii) Discuss if there were elements in the RCT that would be different in a routine application setting
Training in internet and Virtual Coach software would be required for routine application setting.
22-i) Restate study questions and summarize the answers suggested by the data, starting with primary outcomes and process outcomes (use)

Yes. “In this trial, providing overweight participants with access to a Virtual Coach in addition to a pedometer and website appeared sustain step count over the course of the 12 week study, whilst step counts in control participants decreased from the start to the end. The percent change in step count between those in the intervention and those in the control arms, from the start of the study compared to the end of the study did not reach the threshold for significance (p=0.07). However, repeated measures analysis found a significant difference when comparing percent changes in step counts between control and intervention participants over all time points (P = 0.02).”

22-ii) Highlight unanswered new questions, suggest future research

Yes. “we demonstrated a sustained level of activity in overweight participants provided with a Virtual Coach in addition to a pedometer and web-based feedback, compared to a decline seen in those provided with a pedometer and web-based program alone. Further work should examine the long-term benefits of Virtual Coaching and the extension of this application to a wider patient population.”

Other information

23) CONSORT

"ClinicalTrials.gov NCT00792207"

24) CONSORT

Full trial protocol can be accessed from the authors by request. Details available at clinicaltrials.gov.

25) CONSORT

Yes. “Funding Source:Partners IS Research Council”

X26-i) Comment on ethics committee approval

Yes. “The study was reviewed and approved in July 2007 by the institutional review board of the Massachusetts General Hospital.”

x26-ii) Outline informed consent procedures

Yes. Paper consent was used. “Sealed, ordered envelopes were prepared containing information about group assignment and only opened after the participant consented.”

X26-iii) Safety and security procedures

Risks of participation were very limited but were outlined in the consent form.

X27-i) State the relation of the study team towards the system being evaluated

Yes ‘The authors have no relevant conflicts of interest to disclose”
